# CRISPR/Cas therapeutic strategies for autosomal dominant disorders

**DOI:** 10.1172/JCI158287

**Published:** 2022-05-02

**Authors:** Salvatore Marco Caruso, Peter M.J. Quinn, Bruna Lopes da Costa, Stephen H. Tsang

**Affiliations:** 1 Department of Biomedical Engineering and; 2 Jonas Children’s Vision Care and Bernard and Shirlee Brown Glaucoma Laboratory, Columbia University, New York, New York, USA.; 3 Edward S. Harkness Eye Institute, NewYork-Presbyterian Hospital, New York, New York, USA.; 4 Institute of Human Nutrition, Department of Ophthalmology and Department of Pathology and Cell Biology,; 5 Columbia Stem Cell Initiative, and; 6 Vagelos College of Physicians and Surgeons, Columbia University, New York, New York, USA.

## Abstract

Autosomal dominant disorders present unique challenges, as therapeutics must often distinguish between healthy and diseased alleles while maintaining high efficiency, specificity, and safety. For this task, CRISPR/Cas remains particularly promising. Various CRISPR/Cas systems, like homology-directed repair, base editors, and prime editors, have been demonstrated to selectively edit mutant alleles either by incorporating these mutations into sgRNA sequences (near the protospacer-adjacent motif [“near the PAM”]) or by targeting a novel PAM generated by the mutation (“in the PAM”). However, these probability-based designs are not always assured, necessitating generalized, mutation-agnostic strategies like ablate-and-replace and single-nucleotide polymorphism editing. Here, we detail recent advancements in CRISPR therapeutics to treat a wide range of autosomal dominant disorders and discuss how they are altering the landscape for future therapies.

## Current therapeutic editing landscape and technologies

Driven by discoveries in molecular biology and genomics, bench-to-bedside researchers now have the resources to develop potent gene therapies for a wide range of inherited disorders. Tremendous progress has been made in the treatment of autosomal recessive disorders, as these conditions can be targeted by the unique therapeutic approach of gene supplementation, where delivery of exogenous genes can restore healthy biological function. This trend is highlighted by the recent boom in clinical trials of gene therapies, including the FDA-approved voretigene neparvovec (Luxturna), the first approved therapy for RPE65-linked retinal dystrophy (1–6). Unfortunately, autosomal dominant (AD) disorders, where only one mutant allele is required to drive disease progression, do not always respond to similar strategies, particularly when mutant proteins misfunction, gaining deleterious effects (negative gain of function) (7).

Several technologies have been extensively studied for their abilities to treat AD disorders, such as small interfering RNAs (siRNAs), short hairpin RNAs (shRNAs), antisense oligonucleotides (ASOs), transcription activator–like effector nucleases (TALENs), zinc-finger nucleases (ZFNs), and strategies based on clustered regularly interspaced short palindromic repeats (CRISPR) (Table 1). These strategies, however, have unique situational limitations. For example, patisiran, a siRNA for the treatment of transthyretin (ATTR) amyloidosis, has been approved by the FDA but requires repeat dosage every 3 weeks (8). Meanwhile, ASOs can achieve exon skipping in Duchenne muscular dystrophy, but the protein must function in its shortened form, limiting applications (9, 10). Ideal therapeutics for AD disorders can correct pathology at the genomic scale in a manner that is both mutation-agnostic (capable of addressing multiple disease-linked mutations) and allele-specific (able to distinguish between diseased and healthy alleles). CRISPR-associated (Cas) endonuclease editing has quickly emerged as a front-runner in therapeutics because of its versatility, efficiency, and sustained effects, resulting in applications of CRISPR/Cas for the in vivo treatment of ATTR and amyotrophic lateral sclerosis (ALS) (11–13).

**Figure 1:**
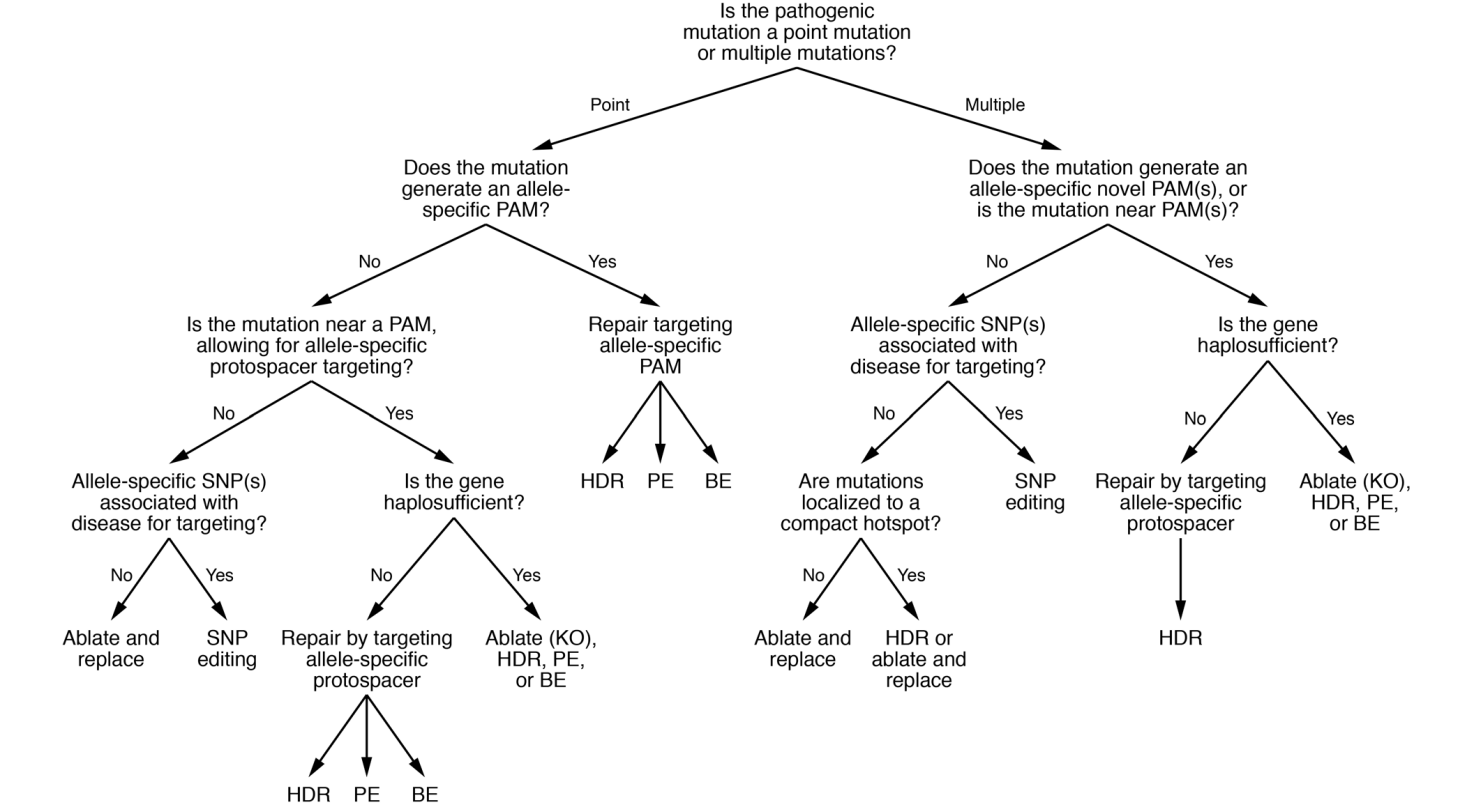
CRISPR/Cas road map for the development of autosomal dominant therapeutics. Decision-making tree that allows researchers to determine the most appropriate therapeutic editing strategy based on responses to a series of questions. This decision tree is particularly for dividing cells and requires substantial amendments for adaptation to nondividing cells, including the removal of HDR and the inclusion of alternative approaches such as homology-independent targeted insertion (HITI) and precise integration into target chromosome (PITCH). It is also important to note that this tree is not exhaustive and parallel decisions must also be considered, such as off-targeting specificity and vector cargo limitations. Note: When deciding which CRISPR-based technology to use, it is important to evaluate each experimental design individually. Critical considerations include in vivo delivery strategies and delivery capacities, off-targeting rates, and editing efficiencies.

Advancements in precise CRISPR-based genome engineering tools such as base and prime editors have played critical roles in increasing safety and efficiency. Similarly, Cas variants have freed CRISPR systems from stringent protospacer-adjacent motif (PAM) dependencies and packaging limitations, vastly expanding the potential for translational approaches. This Review details recent advancements in CRISPR-based strategies to correct a wide range of AD disorders and explores their potential future directions. Furthermore, we highlight the impact of these advancements on the decision-making process in the development of precision therapeutics, providing a road map for strategy selection (Figure 1).

## CRISPR/Cas gene editing techniques in AD disorders

CRISPR/Cas editing strategies are rooted in two key components: a Cas endonuclease engineered to induce double-strand breaks (DSB) or single-strand DNA breaks (SSB), and a single guide RNA (sgRNA) to direct the Cas enzyme to the target site (14). These sgRNAs are designed to complement the targeted DNA sequence — the protospacer — and are spatially conserved relative to a PAM. The PAM sequence, essential for enzymatic activity, is dependent on the type of Cas used — *Streptococcus pyogenes* Cas9 (SpCas9) recognizes a 5′-NGG-3′ PAM, where N represents any potential base pair, while *Staphylococcus aureus* Cas9 (SaCas9) recognizes a 5′-NGRRT-3′ PAM, where R is a purine (A or G) (15). While early iterations of CRISPR/Cas systems were used as tools for basic research, the recognition of this technology’s therapeutic potential quickly gave rise to developments such as HDR-mediated editing, base and prime editors, ablate-and-replace strategies, SNP editing, and Cas-mediated transcriptional regulation.

### HDR-mediated editing in corneal dystrophy and epidermolysis bullosa.

In recent years, CRISPR strategies built on homology-directed repair (HDR) have gained considerable traction for their potential as therapeutics. These systems aim to directly edit the sequence back to a healthy WT state by co-delivering a CRISPR/Cas system with an HDR template comprising the desired WT sequence flanked by two arms homologous to the native DNA strand (16–18). By inducing CRISPR/Cas–mediated DSBs, the endogenous DNA repair mechanisms can recognize the homologous regions of the HDR template as native and model the repair process on this healthy template. Both single CRISPR– and dual CRISPR–induced DSBs can mediate this process; the latter have been demonstrated to offer better efficiency for installing larger HDR templates (19). Compared with non-homologous end joining (NHEJ), the HDR repair pathway is more precise and offers lower indel frequency, but remains less efficient in postmitotic cells, potentially limiting translational applications (20). Consequently, ex vivo therapies that allow for clonal selection and reinfusion may benefit most from this strategy. Despite these shortcomings, critical developments regarding CRISPR/Cas designs have produced several clinical trials dependent on HDR to treat various AD disorders.

Granular corneal dystrophy (GCD) is an AD disorder characterized by irregular granular opacity depositions in the corneal stroma. To treat GCD linked to the TGF-β–induced (*TGFBI*) gene, Taketani et al. implemented an in vitro single-stranded oligodeoxynucleotide (ssODN) HDR template system reliant on a Cas9/sgRNA–mediated DSB targeting the R124H mutation in TGFBI (21). By incorporating the R124H mutation in the spacer sequence of the sgRNA, this 1–base pair difference can allow the system to distinguish between mutant and WT alleles, directing Cas9 cleavage to the mutant allele. The allele specificity of a 1–base pair mismatch has been previously demonstrated in rat embryonic fibroblasts, human fibroblasts, and induced pluripotent stem cells (22–24). Furthermore, ssODNs have been shown to offer better editing than their double-stranded counterparts. Using an ssODN template, Taketani et al. achieved 20.6% editing efficiency in vitro in heterozygous and 41.3% in homozygous mutations with no reported off-target effects in sites predicted to be most susceptible based on sequence similarity (21).

The strategy described above, also called “near the PAM,” can be highly beneficial in designing allele-specific systems but bears several caveats. First, the mutant base pairs must be within proximity of a suitable PAM site. Second, single–base pair mismatches between the mutant and WT alleles may not be enough to always prevent cleavage of the WT allele, which can trigger undesired NHEJ and indels (25). Therefore, each potential therapeutic requires screening of the surrounding genome for viable PAM sites as well as allele specificity, neither of which is guaranteed. A similar approach for designing allele-specific systems is to design the CRISPR/Cas system around a unique PAM generated by the mutation. This approach, called “in the PAM,” has been used by Courtney et al. to achieve allele-specific, HDR-mediated editing via a novel PAM generated by a KRT12 mutation (26). However, like the near-the-PAM strategy, in-the-PAM relies on the probability of the mutation generating a unique PAM. Furthermore, the PAM must be unique compared with the native sequence to prevent any enzymatic cleavage of the WT allele. For example, a native NAG sequence converted to NGG by a point mutation can still result in cleavage of the native NAG strand by SpCas9, as Kleinstiver et al. demonstrated (27).

While the development of ssODNs as templates has increased HDR editing efficiency in recent years, efficiency remains relatively low. As HDR occurs in the G_2_ and S phases of the cell cycle, this approach is most applicable to ex vivo studies or in vivo in highly proliferative cell niches. Furthermore, template delivery in vivo remains a noteworthy hurdle in its translation. Off-target effects remain a considerable safety concern with DSB approaches, and high levels of adeno-associated virus (AAV) integration have been observed in Cas9-induced DSBs (28, 29). Therefore, DSB-independent iterations of CRISPR/Cas technology that exhibit high editing efficiencies and have better safety profiles need to be extensively evaluated as potential therapeutics (30).

### Editors for improved precision in AD disorders.

**Figure 2:**
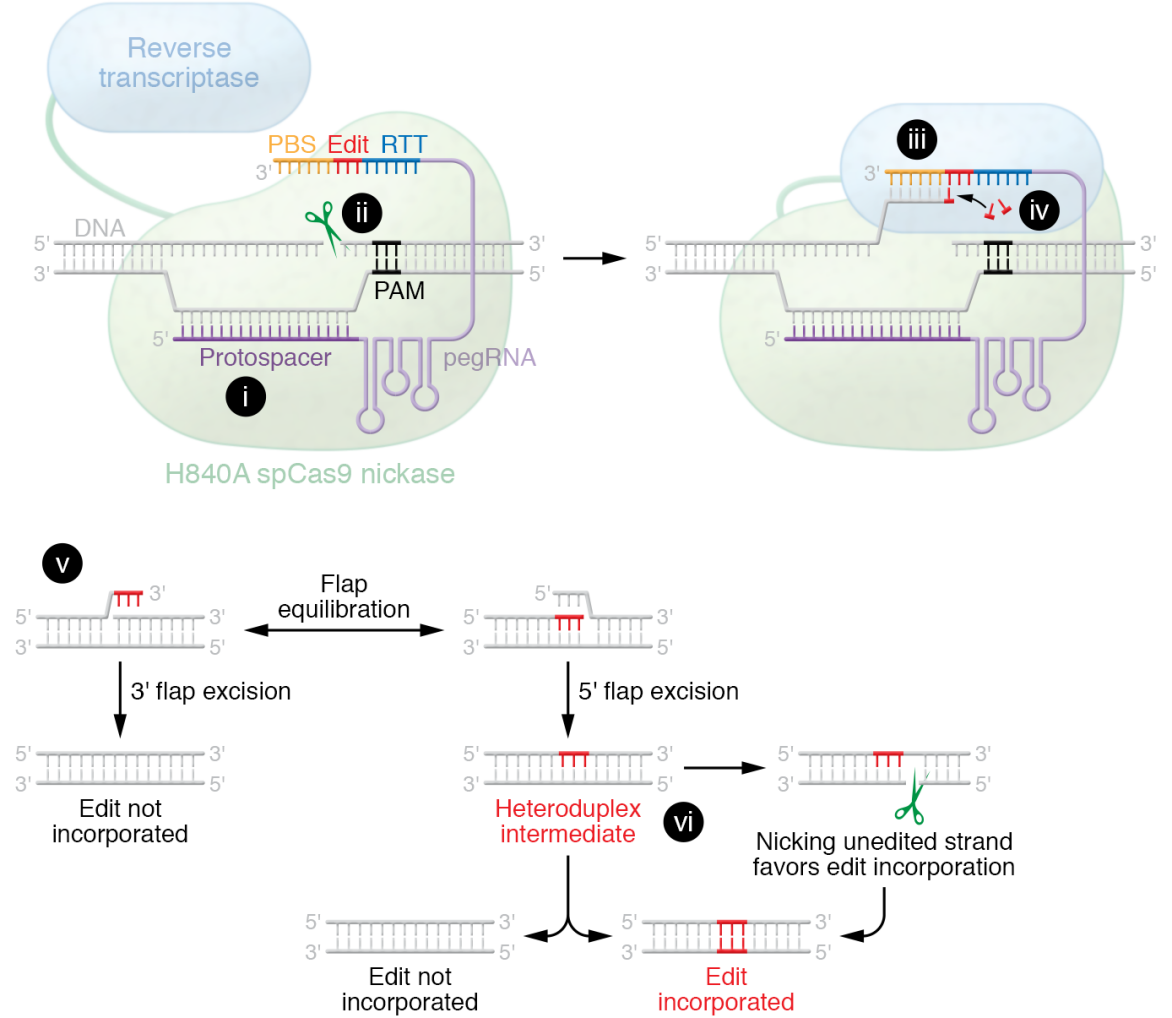
Mechanism of prime editing. Schematic detailing critical steps of prime editing mechanism of action broken down by Cas9 activity, RT activation and function, stochastic endogenous repair mechanisms, and potential editing outcomes. (i) Protospacer hybridization between pegRNA and target DNA sequence. (ii) Engineered SpCas9 creates single-strand break in strand opposite pegRNA hybridization. (iii) Hybridization between the protospacer binding sequence (PBS) of pegRNA and newly generated 3′ flap from nickase activity. (iv) Reverse transcriptase adds nucleotides to the new 3′ end of the nicked DNA strand as directed by reverse transcription template (RTT) found adjacent to the PBS sequence. (v) An equilibrium is achieved between the unedited and edited flaps, where only one is reinserted back into DNA via endogenous DNA repair mechanisms. (vi) Insertion of 3′ flap back into the DNA and pruning of the 5′ flap by exonucleases results in the formation of a heteroduplex, where mismatch repair mechanisms determine whether the unedited strand will be remodeled in response to the edit, or whether the edit will be undone with the unedited strand as template. This process can be shifted in favor of incorporating the edit by introducing an sgRNA that nicks the unedited strand, increasing mismatch repair and improving editing efficiencies. Adapted from da Costa et al. (30).

Base and prime editing are novel DSB- and cell cycle–independent genome editing tools. Both methodologies lead to higher editing efficiency than HDR and have improved safety profiles due to their being DSB-free methodologies. There are two established classes of base editors: cytosine base editors (CBEs) and adenine base editors (ABEs) (31, 32). CBEs are the fusion of a Cas9 nickase (an engineered variant capable of SSBs) with a deaminase and a uracil glycosylase inhibitor, allowing for C→T base pair conversion. ABEs are the fusion of a catalytically dead Cas9 or a Cas9 nickase with two tRNA adenine deaminases (TadA), capable of facilitating A→G transversions. Prime editing is based on the use of an optimized Moloney murine leukemia virus (MMLV) reverse transcriptase fused to an SpCas9 nickase, H840A, guided by a prime editing guide RNA (pegRNA), as shown in Figure 2. Unlike base editing, prime editing can install all possible transitions and transversions in addition to small insertions and deletions. While prime editing may have a slightly higher indel rate, it has a substantial increase in flexibility, does not lead to bystander mutations, and is less reliant on ideal PAM positioning (33). For an in-depth overview of base and prime editing, see recent reviews (30, 34, 35).

Previously, base editing has been used in vivo to ameliorate an AD model of Hutchinson-Gilford progeria syndrome (HGPS). A rare genetic disorder, HGPS is characterized by accelerated aging and a short lifespan. Lentivirus-mediated delivery of ABE efficiently corrected patient-derived fibroblasts, leading to restoration of normal splicing, reduced levels of the progerin protein, and reestablishment of nuclear morphology (36). Furthermore, no off-target DNA or RNA editing was detected using the *LMNA*-targeting sgRNA and ABE7.10max-VRQR editor. To show the translatability of their work, Koblan et al. used dual AAV9-mediated delivery of a split-intein base editor and the *LMNA*-targeting sgRNA into a mouse model of progeria (36). Retro-orbital injection of the AAV base editing therapeutic at postnatal day 14 extended median lifespan from 215 to 510 days, while treated progerin mice had ameliorated *LMNA* transcript mis-splicing, reduced levels of progerin protein, improved aortic pathology, and increased vitality (36). Meanwhile, Lim et al. have also applied CBEs via a split-intein approach to introduce nonsense mutations and render the *SOD1* gene in ALS disabled, achieving high editing efficiencies in spinal cord cells successfully transduced and expressing the full editor (37). Since prime editing is still a nascent technology, in vivo treatment of AD disorders is limited. However, prime editing has already been applied for the development of several in vitro and in vivo disease models, including PPP2R5D-associated intellectual disabilities and STAT1-associated immune disorders (38, 39). With this technology, Lin and colleagues developed a mouse model of dominant cataract disorder bearing a pathogenic deletion in exon 3 of *Crygc*, editing N2A cells derived from this model back to healthy states with prime editing. The researchers observed efficiencies as high as 33.3% and provided evidence of prime editing’s therapeutic capabilities (40).

Editors have also been applied in vivo for the editing of RNA, an attractive alternative to DNA editing owing to decreased off-target events and better safety profiles (41). Developed by Cox et al., RNA Editing for Programmable A to I Replacement (REPAIR) relies on the fusion of a dead Cas13b (dCas13a) to the ADAR2 domain of adenosine deaminase. In cells transfected with cDNA encoding mutant FANCC protein linked to autosomal dominant Fanconi anemia, researchers reduced mutant transcript levels by 23% (42). Similarly, a cytosine RNA editor called RESCUE was also developed by fusing of dCas13 to an evolved ADAR2 capable of cytidine deaminase activities (43). While both techniques require further optimization for clinical relevance, their transient and reversible effects, which make them safer candidates, also reduce their therapeutic potential, as long-term editor expression is essential for maintaining therapeutic effects at the transcriptomic level, which raises immunogenic concerns and requires repeat dosing. Consequently, in vivo evaluations of this technology, especially for AD disorders, are considerably lacking.

Though base and prime editors are the new iterations of CRISPR/Cas systems, considerable efforts are being made to improve them. More recently, base editors that can install select transversion mutations (C→A and C→G point mutations) have been developed, expanding their use for treating AD disorders (44–46). Further, a series of teams developed dual-function base editors that combined the functions of adenine and cytosine base editors, allowing for simultaneous introduction of C→T and A→G mutations (47–49). Despite high editing efficiency with minimal off-target effects, base and prime editors remain restricted by their mutation-specific approach. However, multiplexed or simultaneous CRISPR/Cas9 editing is safe and feasible and has been applied to DSB-independent approaches (48, 50). A new but exciting technological advancement, twin prime editing, can introduce large sequences of DNA in a specific manner (51). Furthermore, HDR efficiency has been shown to decrease with increasing template size, while delivery of increasingly large templates remains a translational challenge (52).

### Ablate-and-replace CRISPR/Cas in retinitis pigmentosa.

To develop a more ideal therapeutic capable of addressing all pathogenic mutations independent of their proximity to a PAM, researchers have begun to employ an “ablate and replace” strategy. In this method, both the mutant and WT alleles are ablated via CRISPR/Cas9–induced NHEJ and replaced by the co-delivered, healthy CRISPR-resistant cDNA to support biological function (53). Unlike with previous in-the-PAM and near-the-PAM strategies, the sgRNA for this system need not be allele specific, but it must target an exon to ensure reading frame shift and disruption. Meanwhile, to ensure that the native gene ablation occurs while preserving the healthy cDNA, silent mutations are typically introduced in the cDNA. The addition of silent mutations is designed to alter either the PAM site or the protospacer sequence targeted by the guide RNA(s) required for gene ablation, as demonstrated by Paquet et al. in human stem cells (54). It is important to note that this silent-mutation design consideration can be applied to HDR and editor strategies as well to prevent subsequent cleavage and undesired indel formation.

One application of this strategy is used to treat AD rhodopsin-linked retinitis pigmentosa (RP). Rhodopsin, a light-sensitive G protein–coupled receptor, is critical for photoreceptor function and has been associated with several AD disorders (55–58). To treat mutations in rhodopsin in a mutation-agnostic way, Tsai et al. developed an ablate-and-replace strategy that relies on a dual AAV system to deliver the CRISPR-resistant cDNA and two guide RNAs targeting exon 1 of the rhodopsin gene, *RHO* (59). To maximize safety, one AAV delivers the Cas9 sequence while the other AAV co-delivers the two sgRNAs and humanized rhodopsin cDNA, ensuring that ablation only occurs in the presence of gene supplementation. To make the cDNA CRISPR-resistant, silent mutations were introduced into the PAM sites targeted by the sgRNAs, preventing Cas cleavage (59). In *RHO^P23H/P23H^*, *RHO^P23H/+^*, and *RHO^D190N/+^* mouse models of RP, researchers demonstrated a substantial preservation of outer nuclear thickness in treated eyes and functional rescue via electroretinography. Endogenous mouse mRNA levels were downregulated by ablation alone, as determined by quantitative PCR, while human *RHO* mRNA levels were upregulated under cDNA delivery. Transient mRNA levels were measured instead of protein as commercial antibodies specific to mice and humans are not available. Recently, Wu et al. also treated a novel humanized mouse model of C110R rhodopsin-linked RP with ablate-and-replace, establishing further proof of concept and highlighting this strategy’s ability to treat multiple pathogenic mutations with a single therapeutic (60).

As with editors and HDR, the ablate-and-replace strategy has limitations and situational shortcomings. First, ablate-and-replace requires gene supplementation, which may not always be feasible given the sizes of various genes and packaging limits of common vectors such as AAVs and lentiviruses. Second, since the endogenous healthy allele is being ablated, long-term cDNA expression is imperative. However, several studies have shown that cDNA expression may decrease with time, particularly in dividing cells (61, 62). Furthermore, overexpression of transgene may prove toxic and does not allow for multiple alternatively spliced variants. Lastly, ectopic expression of the supplemented protein may be problematic, as Pellissier et al. demonstrated in expressing hCRB1-A protein in AAV-transduced mouse models (63). As a result, ablate-and-replace is reserved for genes small enough for augmentation and sufficient promoter flexibility, is best applicable to nondividing cells, and must have its cDNA components evaluated for long-term expression. While FDA regulations are more stringent in the case of integrating therapeutics, host genome integrating approaches to ensure sustained genomic supplementation may prove pivotal in future clinical efforts tackling diseases in dividing cells.

### SNP editing in Huntington’s disease and severe congenital neutropenia.

While the ablate-and-replace strategy has greater versatility than in- and near-the-PAM CRISPR/Cas designs, the translational aspect of this therapeutic is strongly handicapped by cDNA delivery size constraints and long-term expression concerns. Consequently, selective ablation of the mutant allele while the healthy allele is left intact remains an attractive approach. Naturally occurring heterozygous single-nucleotide polymorphisms (SNPs) have recently become high-priority targets for designing allele-specific strategies. While these SNPs may not be disease-causing in nature, SNPs tightly associated with disease-causing mutations can create allele-specific protospacer sequences and PAM sites akin to in-the-PAM and near-the-PAM designs. For example, in keratin 12–associated (KRT12-associated) Meesmann epithelial corneal dystrophy, Courtney et al. targeted a unique PAM generated by a SNP to selectively ablate the mutant allele, inducing NHEJ in 38.5% of the clones sequenced (26). In designing SNP editing strategies, however, it is important to note that the position of the mismatch in the protospacer can have substantial impact on allele specificity, as mismatches in the 5′ ends of guide RNAs can be well tolerated and result in cleavage of the healthy allele, as demonstrated by Fu et al. (64).

**Figure 3:**
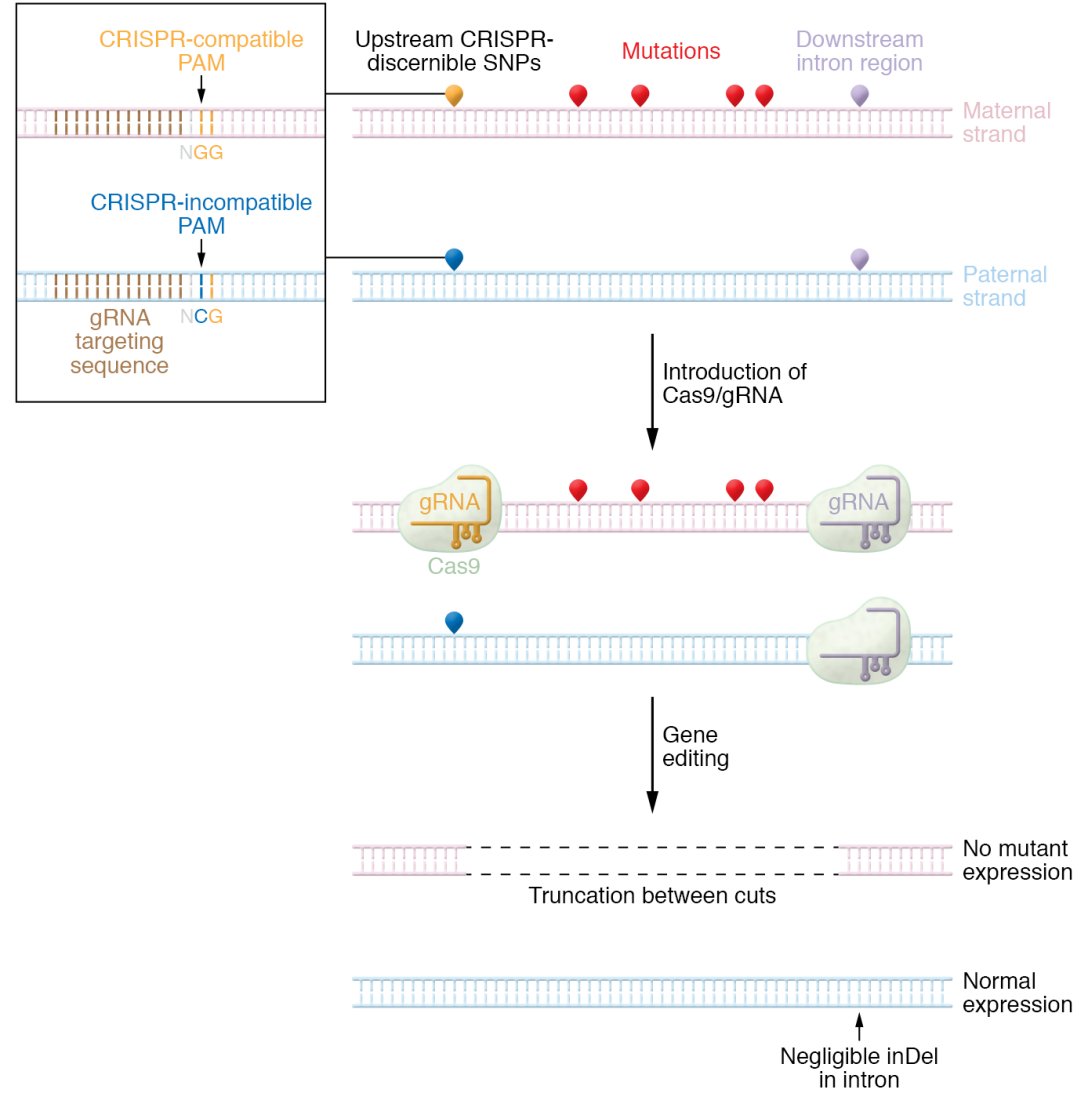
SNP editing for the mutation-agnostic, allele-specific treatment of autosomal dominant disorders. SNP editing has the potential to treat multiple mutations with a single therapeutic, relying on the selective ablation of the mutant allele by targeting CRISPR/Cas systems to allele-specific SNPs. Upstream and downstream SNPs flanking regions of high pathogenic mutations can be used to selectively create CRISPR/Cas systems and induce a targeted deletion that ablates allele expression.

To improve efficiency, a pair of guide RNAs targeting different SNPs can induce a targeted gene deletion by NHEJ that renders the mutant allele useless while preserving its WT counterpart (65). A detailed mechanistic schematic is provided in Figure 3. To highlight the potential applications of this SNP editing in AD disorders, Monteys et al. detailed a dual sgRNA CRISPR/Cas9 system for the treatment of Huntington’s disease (66). By screening nearly fifty 5′ and 3′ SNPs flanking exon 1 of the human *HTT* gene, researchers developed a pair of sgRNAs that exhibited high allele specificity, minimal off-target effects, and robust efficiency in vitro. This system was further validated in a transgenic Huntington’s disease mouse model bearing mutations in the *HTT* gene; a substantial decrease in HTT mRNA and protein was observed in the right hemisphere of the brain following a localized viral injection compared with the contralateral, untreated hemisphere (67).

Similarly, Christie et al. have presented an allele-specific strategy for the targeted deletion of autosomal dominant TGFBI corneal dystrophies. While targeting a heterozygous 5′ SNP to differentiate between alleles, they used a shared intronic 3′ sgRNA to minimize the size of the targeted deletion and maximize deletion rates. As the region targeted by the 3′ sgRNA is intronic, deleterious effects of NHEJ-induced reading frame shifts in the WT allele are mitigated and are expected to be well tolerated (67). With this system, Christie and colleagues demonstrated the allele specificity of their 5′ sgRNA (up to 99% of NHEJ linked to the correct allele) and successful targeted deletion in patient-derived lymphocyte cell lines, providing a foundation for future therapeutic SNP editing approaches. SNP editing has also been successfully applied in vitro to ameliorate mutations in the *ELANE* gene linked to severe congenital neutropenia. These predominantly AD disorders have been previously demonstrated to cause defects in cell survival and maturation in bone marrow hematopoietic stem cells that are rescued under CRISPR/Cas9 knockout of *ELANE*, as demonstrated by Makaryan et al. (68). Since SNPs flanking *ELANE* are limited, Sabo et al. elected to use a 3′-UTR SNP-specific sgRNA (rs1683564) in combination with a non-allele-specific sgRNA targeting intron 4 (69). Here, the researchers confirmed that allele-specific editing corrected cellular abnormalities and increased proliferation by 41% in CD34^+^ cells, providing further evidence of therapeutic SNP editing.

While there is immense potential for these SNP editing strategies to be applied to translational therapeutics, SNP editing has limitations. Like in- and near-the-PAM strategies, SNP editing also suffers in its versatility as a result of its chance reliance on proximity to a PAM site or the creation of a novel PAM. SNP prevalence in patient populations will also impact the number of patients suitable for treatment. There is also no assurance that dual cleavage will consistently result in targeted regional deletion and it must be screened for each pair of sgRNA candidates. As this is a selective ablation strategy, the gene must be haplosufficient, allowing the remaining allele to maintain biological function. Lastly, it is important to note that this methodology relies on the genetic sequencing of the patient to ensure that he or she bears the targeted SNP(s), and determining the linkage of SNPs and mutations (either in *cis* or in *trans*) may prove problematic.

### dCas9 fusions for transcriptional regulation in early-onset obesity.

While the methodologies discussed so far rely on direct editing of DNA or RNA, alternative approaches that regulate transcription without the introduction of edits or genomic breaks have inherently safer profiles. As a result, CRISPR/Cas systems have been explored for their ability to regulate gene expression. These systems consist of a standard sgRNA and a catalytically inactive dCas9 fused to transcriptional or chromatin effector domains responsible for regulating the targeted gene(s). Two main approaches are widely reported: the use of effectors to modify chromatin structure to increase or decrease accessibility and thus increase or decrease expression, and the use of transcription activators or repressors to modulate expression accordingly.

In AD disorders characterized by haploinsufficiency, CRISPR activation (CRISPRa) or the upregulation of gene expression offers a unique therapeutic approach. Using CRISPR-dCas9 fused to a VP64 transcriptional activator domain, Maeder et al. demonstrated this technology’s ability to upregulate several genes simultaneously and synergistically in vitro (70). Unlike gain-of-function mutations, loss-of-function mutations in haploinsufficient genes associated with AD disorders can benefit from non-allele-specific activation. Using CRISPRa systems targeting both the promoter and enhancer of *SIM1* in an AD mouse model of obesity, Matharu et al. observed efficient and specific upregulation of *SIM1* mRNA levels as well as a statistically significant reduction in mouse weight (71). Here, nonspecific amplification has clear therapeutic effect, and its freedom from allele-specific strategies allows for wider potential application. Furthermore, this system has the potential to treat polygenic disorders and can be used to regulate entire gene networks, as initially shown by Maeder et al. and reproduced by Bester et al. (70, 72). Meanwhile, CRISPR interference (CRISPRi), developed by Qi et al., or the downregulation of gene expression can be an effective tool for disrupting transcriptional activation and suppressing gene expression (73). Like SNP editing or in/near-the-PAM approaches, a differentiable SNP or mutation can be targeted to achieve allele-specific interference and downregulate the mutant gene, as explored by Mandegar and colleagues (74). Having previously been demonstrated to have a lower tolerance for mismatches than Cas9 nuclease, CRISPRi has the potential to better discern between healthy and mutant alleles, necessitating in vivo evaluations for AD diseases (75).

A key advantage of CRISPRa is the ability to upregulate expression of genes that are simply too large for traditional gene augmentation approaches. While either approach is an option for smaller haploinsufficient genes, genes that surpass packaging limits for cDNA delivery are great targets for therapeutic exploration. Furthermore, CRISPRi has the distinct advantage of reversible suppression compared with catalytically active CRISPR/Cas9 therapies. However, both technologies have several limitations. While allele-specific designs can be generated for AD disorders, transcriptional regulation also relies on the probability-driven presence of a suitable PAM site or SNP prevalence. Furthermore, long-term expression of the Cas9 enzyme is essential for sustained therapeutic effect and may be problematic given heavily debated concerns regarding long-term immunogenicity (76–79). Lastly, efficacies of these strategies tend to be lower than those of enzymatically active Cas9 nuclease approaches, demanding substantial optimization (80, 81).

## The future of CRISPR/Cas strategies for AD disorders

Although these various CRISPR/Cas systems are distinguished by their situational advantages, the Cas enzyme remains a common denominator. Recent advancements in Cas variants have revolutionized CRISPR/Cas designs in AD disorders. While these enzymes are often selectively applied for their unique functional benefits, size limitations driven by vector capacities have become a major consideration in translational CRISPR/Cas systems. Consequently, many efforts are focused on increasing the delivery efficiency and cargo capacity of vectors. While achieving more efficacious CRISPR/Cas systems through novel compact designs and innovative vectors, researchers have also made substantial progress in improving the safety of these systems via minimization of off-target effects, self-terminating designs, and more.

### Cas variants and expansion beyond PAM restrictions.

While the preferred CRISPR/Cas variant today is widely considered to be SpCas9, various orthologs have gained attention in recent years due to their potential advantages. SaCas9, for example, is a compact ortholog of SpCas9 that targets an NNGGGT PAM, unlike SpCas9’s NGG (82). Similarly, NmCas9, derived from *Neisseria meningitidis*, targets 5′-NNNNGATT-3′, which is believed to offer greater specificity because of the additional PAM sequence complexity (83). A plethora of engineering, like the directed evolution of Cas9, has also gone into modifying these enzymes, resulting in products such as eSpCas9 — an enhanced nuclease with a charged groove to stabilize DNA strands, allowing for greater specificity and fewer off-target effects (84). Similarly, SpCas9-VQR, xCas9, and many more innovations are the direct products of directed evolution. These variants and their characteristics are summarized in Table 2.

While optimizing the efficiency and specificity of Cas has always been a major priority, these innovations are all still limited by Cas-specific PAM restrictions. Despite the growing list of PAM targets giving researchers greater versatility and access to the human genome, regions remain inaccessible by CRISPR. Consequently, Walton et al. worked to expand these Cas enzymes beyond their PAM restrictions, characterizing a nearly PAM-less Cas9 variant. Using structure-guided engineering, the researchers developed a potent SpG Cas variant capable of targeting an NG PAM with high fidelity (85). Similarly, this group also produced an even more generalized Cas variant — SpRY — that is capable of inducing highly efficient NRN-targeted breaks (R representing purines A and G) with less efficient cleavage at NYN PAMs (Y representing pyrimidines C and T), approaching a nearly limitless PAM variant (85). From a predetermined list of potential off-target sites, researchers observed a modest increase in off-targeting activity that is in line with historical WT SpCas9 off-targeting levels. While subsequent studies must be done to analyze whole-genome off-targeting, it is possible that the specificity provided by a 20–base pair spacer may mitigate additional off-target effects expected when relaxing the PAM and increasing the number of potential interactive sites.

In selecting Cas variants for therapeutic applications, several key considerations must be made in therapeutic design: namely, on rate; PAM sequence; specificity; and variant cDNA size. The optimal Cas variant for treating AD disorders and beyond would be a compact, potent, highly specific, and unrestricted enzyme capable of editing the entire human genome. These innovations expand the versatility of CRISPR and bridge the gap between therapeutics and genomic engineering.

### Developments in vector technologies.

While creating more potent enzymes and expanding beyond traditional PAM restrictions, researchers have also developed vectors capable of efficiently delivering increasingly large cargoes for these CRISPR/Cas systems with greater specificity. While designs can prove highly efficacious in vitro, localized and effective in vivo delivery remains a limitation for many systems. Consequently, methodologies such as directed evolution have become widely implemented to develop novel vectors, viral and nonviral, capable of addressing these issues.

Using directed evolution across multiple species, Tabebordbar et al. synthesized MyoAAV, a novel RGD motif–bearing AAV capable of potent muscle-directed gene delivery (86). By first preparing a viral capsid library containing various structural mutations and motifs with unique identifying payloads, the researchers performed subsequent rounds of in vivo injections (directed evolution) in mice and nonhuman primates and harvested capsids from tissues, amplifying genomic identifiers to delineate subpopulations of best-transducing capsids. From these efforts, MyoAAV and several other candidates were identified for their potential to deliver gene editing cargoes aimed at treating AD disorders like muscular dystrophy. This methodology has been expanded to a wide range of applications, such as increasing retinal tropism, improving hepatocyte specificity, and more for both viral and nonviral vectors (86–88). Novel AAVs for improved specificity and transduction efficiency, such as EC71, MV50, and NHP#26, remain critical points of innovation for translating CRISPR therapeutics into the clinic (89–91).

While Cas variants can potentially decrease payload size, some systems and editors remain too large for AAV vectors despite engineering efforts, which remain capped at approximately 4.5 to 5 kb. However, dual AAV strategies using split-intein base and prime editors have shown good in vivo efficacy (36, 37, 92–94). Additionally, non-integrating lentiviral vectors have quickly risen in popularity due to their larger cargo capacity of approximately 8 kb, best demonstrated by the development of a lentiviral vector delivering ALD protein in X-linked adrenoleukodystrophy (95–97). Moreover, while the concerns regarding genotoxicity and oncogenesis in integrating lentiviral vectors add a unique FDA regulatory challenge, integrating vectors have the unique advantage of long-term expression sustained through cell division, which may be highly attractive for treating proliferative niches. However, editors may be at the packaging limits for these vectors and therefore may limit clinical translatability owing to decreased titers.

Consequently, nonviral vectors capable of delivering large payloads with high efficiency and low toxicity have become attractive candidates, especially for ribonucleoprotein (RNP) delivery, which has been shown to have fewer off-target effects (98, 99). Lipid nanoparticles (LNPs) capable of delivering both the Cas9 protein and guide RNA(s) required for therapy have been demonstrated by Finn et al. to achieve robust delivery in vivo, ablating the mouse transthyretin gene in liver and reducing serum protein levels by over 97% for at least 12 months (100). Despite its high efficiency, researchers observed detectable levels of off-targeted editing in peripheral tissues — a key concern for LNPs given their broad tropism. To combat this issue, engineered virus-like particles (eVLPs) have been developed to overcome specificity bottlenecks while also increasing cargo capacities. Banskota et al. recently presented an in vivo eVLP-mediated base editing approach for the successful reduction of PCSK9 protein levels and partial restoration of vision in two separate mouse models (101).

### Safety considerations in CRISPR/Cas systems.

As is made evident by lentiviral integration concerns and FDA responses, safety must be an ultimate priority of CRISPR therapeutics. While several studies have shown short-term Cas expression safety, long-term expression and potential immune responses remain heavily debated (76–79). Consequently, CRISPR/Cas safety designs have evolved to meet these needs, including concepts such as transient RNP-mediated delivery, cell type–specific promoters, self-terminating systems, time-delayed safety switches, and modified Cas variants.

To create a transient expression profile of Cas9, Li et al. inserted a CRISPR-sensitive region into the AAV backbone targeted by their sgRNA delivered for in vivo gene editing. Researchers demonstrated successful disruption of the AAV genome and a decrease in SaCas9 levels in injected mice with the self-deleting system compared with SaCas9-injected mice without self-termination (102). Furthermore, researchers demonstrated a dose-responsive removal of SaCas9 in their lead target with no observed off-target effect, evaluating the time course of these two characteristics over 4 weeks. In an all-in-one AAV vector, Ibraheim et al. also demonstrated the use of self-inactivating CRISPR/Cas design in vivo for the safe and effective treatment of type I hereditary tyrosinemia and mucopolysaccharidosis type I (103). Self-deletion kinetics, however, are only first order and lack tunability. As a result, computational biologists are looking to develop quantitative approaches to modeling time-delayed safety switches that alter the kinetics of Cas removal to allow for an initial therapeutic burst followed by a quick deletion to mitigate any off-target or immunological effects. One common approach either artificially induces an ultrasensitive relationship between a stimulus and Cas9 protein levels or capitalizes on intrinsic time delays already present in biological circuits. Unfortunately, computational modeling of these systems has lagged other major advancements, leaving room for improvement in coming years.

Another safety feature applicable to all Cas systems is the use of a modified Cas endonuclease that creates two separate SSBs instead of one DSB. To demonstrate this design, Kocher et al. corrected a hotspot mutation in exon 6 of the *KRT14* gene linked to generalized severe epidermolysis bullosa simplex via a dual sgRNA system predicated on Cas9n — a modified Cas9 capable of creating paired nicks on each strand of the target sequence (104). By the creation of two SSBs up to 100 bp apart targeting unique PAMs in opposite strands of intron 7, the likelihood of achieving off-target editing is greatly diminished. Treating patient-derived human keratinocytes harboring this exon 6 hotspot mutation, the researchers observed HDR efficiencies up to 32%, with correction rates of nearly 20%, while observing no off-target effects in a predetermined set of genes most likely to be edited (104).

## Clinical considerations and future directions

Despite a growing arsenal of tools for modulating highly efficient and safe CRISPR therapeutics to treat AD disorders, large regions of the human genome remain inaccessible. From HDR to base and prime editors, each major development is aimed at improving either efficiency, versatility, translatability, or safety. Here, we have detailed the foundation of CRISPR therapeutics and their recent developments in the context of AD disorders. While major advancements have been made to free CRISPR/Cas from stringent PAM dependencies, improved genome accessibility, vector delivery capabilities, and safety remain critical future directions. By identifying these improvements, as well as the current gaps and challenges, we hope we have provided critical background information that researchers can use to bring CRISPR/Cas forward into the clinics and positively impact the quality of health care.

## Figures and Tables

**Table 2:**
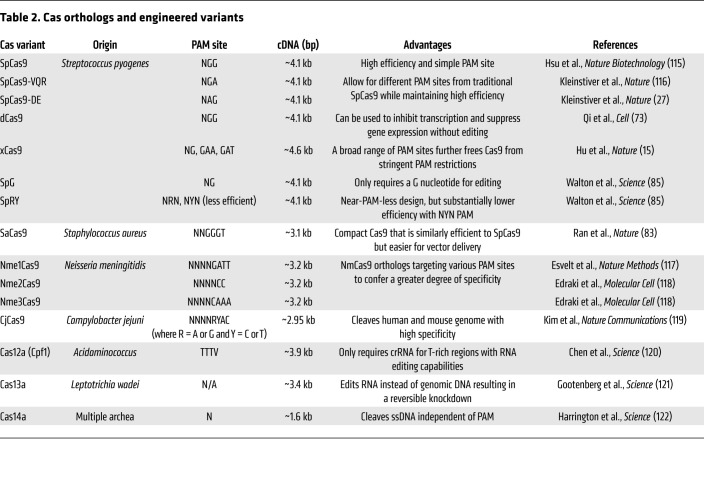
Cas orthologs and engineered variants

**Table 1:**
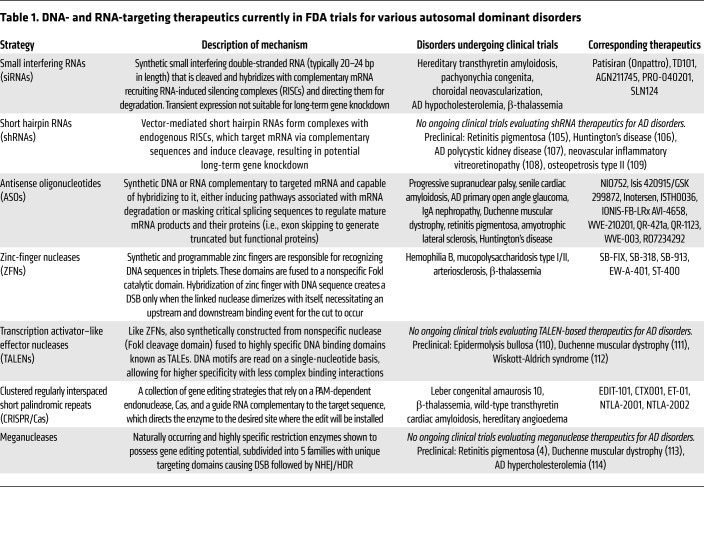
DNA- and RNA-targeting therapeutics currently in FDA trials for various autosomal dominant disorders

## Version 1 05/02/2022

Electronic publication
